# Complete Genome Sequence of Citrobacter freundii Siphophage Sazh

**DOI:** 10.1128/MRA.01317-19

**Published:** 2019-12-12

**Authors:** Whitney L. Crossland, Justin P. Shaw, Chandler O’Leary, Jason Gill, Mei Liu

**Affiliations:** a Center for Phage Technology, Texas A&M University, College Station, Texas, USA; Loyola University Chicago

## Abstract

As an opportunistic pathogen, Citrobacter freundii is involved in a wide spectrum of nosocomial infections. C. freundii phages may prove useful as therapeutics for treating infections caused by multidrug-resistant C. freundii strains. Here, we report the complete genome sequence of C. freundii siphophage Sazh, which is closely related to *Enterobacteria* phages T1 and TLS.

## ANNOUNCEMENT

Citrobacter freundii is a Gram-negative bacterium naturally found in soil, ground water, and the human gastrointestinal tract. As an opportunistic pathogen, C. freundii is involved in a wide spectrum of nosocomial infections (1, 2). Multidrug-resistant strains of C. freundii that carry beta-lactamase, carbapenemase, or other resistance mechanisms have emerged (3–5). Therapies using phages that infect C. freundii may be a viable alternative for treating infections caused by this pathogen.

Phage Sazh was isolated from a municipal wastewater sample collected from College Station, TX, in 2014 using a C. freundii strain as host. LB broth or agar (Difco) was used to culture the host bacterium and phage enrichment at 37°C with aeration. Phage isolation and propagation were conducted using the soft-agar overlay method (6). Sazh was identified as a siphophage using negative-stain transmission electron microscopy performed at the Texas A&M University Microscopy and Imaging Center, as described previously (7). Phage genomic DNA was extracted and purified using a modified Promega Wizard DNA cleanup kit protocol (7). Pooled indexed DNA libraries were prepared using the Illumina TruSeq Nano low-throughput (LT) kit, and the sequence was obtained from the Illumina MiSeq platform using the MiSeq V2 500-cycle reagent kit, following manufacturer’s instructions, producing 597,167 paired-end reads for the index containing the phage Sazh genome. FastQC 0.11.5 (https://www.bioinformatics.babraham.ac.uk/projects/fastqc/) was used to quality control the reads. The reads were trimmed using FASTX-Toolkit 0.0.14 (http://hannonlab.cshl.edu/fastx_toolkit/download.html) before being assembled using SPAdes 3.5.0 (8). Contig completion was confirmed by PCR using primers (5′-AAAAACGCCTAACTTGTCGGTA-3′ and 5′-GCAATGAAACAGGAAGGTGAA-3′) facing off the ends of the assembled contig and Sanger sequencing of the resulting product, with the contig sequence manually corrected to match the resulting Sanger sequencing read. GLIMMER 3.0 (9) and MetaGeneAnnotator 1.0 (10) were used to predict protein-coding genes with manual verification, and tRNA genes were predicted with ARAGORN 2.36 (11). Rho-independent termination sites were identified via TransTermHP (http://transterm.cbcb.umd.edu/). Sequence similarity searches were done by BLASTp 2.2.28 (12) against the NCBI nonredundant (nr), UniProt Swiss-Prot (13), and TrEMBL databases. InterProScan 5.15-54.0 (14), LipoP 1.0 (15), and TMHMM v2.0 (16) were used to predict protein function. All analyses were conducted at default settings via the CPT Galaxy (17) and Web Apollo (18) interfaces (https://cpt.tamu.edu/galaxy-pub).

Siphophage Sazh was assembled at 25.7-fold coverage to a complete genome of 49,665 bp. It has 42.8% GC content, which is lower than that of the host (51.6%) (19). As determined by Emboss Stretcher (20), Sazh shares 85.4% nucleotide sequence similarity with *Enterobacteria* phage TLS (NCBI RefSeq accession number NC_009540) and 64.2% similarity with other T1-like phages, such as Citrobacter phage Stevie (NCBI RefSeq accession number NC_027350) (21). The Sazh genome was opened to follow the same order as *Enterobacteria* phages T1 (NCBI RefSeq accession number NC_005833) and TLS (NCBI RefSeq accession number NC_009540). T1-like proteins (determined by a BLASTp search against the NCBI nr database at an E value of <10^−3^), including those involved in phage morphogenesis and DNA replication, were identified in the Sazh genome. A DNA adenine methyltransferase was also identified, indicating that Sazh likely methylates its DNA-like phage T1 (22). The identified lysis genes include those encoding a holin, an R21-like signal anchor release (SAR) endolysin (23), and a unimolecular spanin.

### Data availability.

The genome sequence of phage Sazh was submitted to GenBank as accession number MH729819. The associated BioProject, SRA, and BioSample accession numbers are PRJNA222858, SRR8761742, and SAMN11191518, respectively.
